# Nitrogen Use Efficiency in Agriculture: Integrating Biotechnology, Microbiology, and Novel Delivery Systems for Sustainable Agriculture

**DOI:** 10.3390/plants14192974

**Published:** 2025-09-25

**Authors:** Bruno B. Navarro, Mauricio J. Machado, Antonio Figueira

**Affiliations:** 1 Department of Genetics, “Luiz de Queiroz” College of Agriculture, University of São Paulo, Piracicaba 13418-900, SP, Brazil; bbachiega@usp.br; 2 Centro de Energia Nuclear na Agricultura (CENA), Universidade de São Paulo, Piracicaba 13416-000, SP, Brazil; mauriciomachado@usp.br

**Keywords:** biofertilizers, microbiome, molecular breeding, nanomaterials, nitrogen transporters, sustainability, symbiosis, synthetic fertilizers

## Abstract

Nitrogen (N) is the primary macronutrient that supports global agriculture. The Haber–Bosch process revolutionized the use of synthetic N fertilizers, enabling significant increases in crop yield. However, N losses from fertilization led to negative impacts on the environment. Improving crops’ N use efficiency (NUE) has been constrained by the limited understanding of N uptake and assimilation mechanisms, and the role of plant–microbe interactions. Among biological approaches, N fixation by cover crops and rhizobia symbioses represents a cornerstone strategy for improving NUE. The adoption of plant growth-promoting bacteria and arbuscular mycorrhizal fungi may enhance N acquisition by increasing root surface, modulating phytohormone levels, and facilitating nutrient transfer. Advances in plant molecular biology have identified key players and regulators of NUE (enzymes, transporters, and N-responsive transcription factors), which enhance N uptake and assimilation. Emerging biotechnological strategies include *de novo* domestication by genome editing of crop wild relatives to combine NUE traits and stress resilience back into domesticated cultivars. Additionally, novel fertilizers with controlled nutrient release and microbe-mediated nutrient mobilization, hold promise for synchronizing N availability with plant demand, reducing losses, and increasing NUE. Together, these strategies form a multidimensional framework to enhance NUE, mitigate environmental impacts, and facilitate the transition towards more sustainable agricultural systems.

## 1. Introduction

Nitrogen (N) is an essential nutrient for plant growth and development, forming the backbone of both amino acids and nucleic acids, as well as chlorophyll, while participating in several primary physiological processes [1]. The plant’s capacity to perceive and respond to changes in soil N availability directly determines plant growth, yield, and survival. Nitrogen is frequently the most limiting nutrient in agricultural soils, and humans have dedicated significant efforts to make it more accessible to plants, shaping agricultural systems throughout history [2]. Approximately half of the world’s population relies on N fertilizers for food security [3], underscoring the critical importance of this element in sustaining modern food systems.

**Figure 1:**
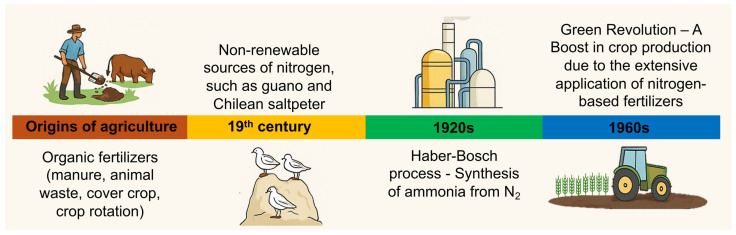
Timeline of nitrogen use in agriculture and its advancements. The figure illustrates key milestones in the history of nitrogen use in agriculture. Initially, organic fertilizers such as manure and animal waste were the primary sources of nitrogen. In the 19th century, non-renewable nitrogen sources, such as guano and Chilean saltpeter, became widely used. The development of the Haber–Bosch process in 1913 revolutionized nitrogen availability by enabling the synthesis of ammonia from atmospheric nitrogen (N_2_). This innovation laid the foundation for the Green Revolution in the 1960s, which saw a significant boost in crop production due to the extensive application of nitrogen-based fertilizers.

Humans have traditionally used organic resources, such as animal and plant waste, to replenish soil nutrients, particularly N, since the beginning of agricultural practices [4]. These practices formed part of an integrated farming system that utilized a self-sustaining nutrient cycle (e.g., crop rotation with legumes), thereby favoring ecological balance and sustaining fertility and food production over extended periods (Figure 1). As the population grew faster, urban areas expanded, and agricultural activities intensified during the 18th and 19th centuries, these resources started to fall short of the increasing food demand. Under this scenario, guano, a N-rich source derived from fossilized seabird excrement found on islands along the Peruvian coast, emerged as a rich and efficient N source [5,6]. Guano was highly valued in international markets for its high N concentration and rapid agronomic response [7]. As America and Europe witnessed a surge in agricultural yield during the 19th century, guano also gained popularity under the label of “white gold.” However, the mismanaged extraction of this non-renewable resource resulted in lower availability and higher prices over time, forcing the search for alternative and sustainable inputs in food production [8].

The pressing demand for increased food production, combined with the guano crisis, stimulated innovation in synthetic N reduction. The “Frank-Caro” process was a groundbreaking achievement in the inorganic fertilizer market. It was developed in the late 1890s, and it involved reacting powdered calcium carbide with nitrogen gas at high temperatures, creating calcium cyanamide fertilizer (Lime nitrogen) as a precursor to ammonia [9]. In the early 20th century, chemists Fritz Haber and Carl Bosch developed a method that revolutionized the modern world [10]. The Haber–Bosch process synthesizes ammonia (NH_3_) from air nitrogen (N_2_) and hydrogen (H_2_) under high pressure and temperature in a more energy-efficient way [10,11]. It became possible to competitively convert N from the atmosphere into a reduced form that plants could use on a large industrial scale. It marked the beginning of the modern agricultural age by enabling the large-scale production of synthetic nitrogen fertilizers and overcoming natural limits to crop productivity [12].

The Green Revolution that occurred in the second half of the 20th century marked a significant milestone in agriculture [13]. This movement led to a significant shift in agriculture with the introduction of high-yielding crop varieties, advances in agricultural machinery, improved irrigation methods, and the increased use of chemical inputs, particularly synthetic fertilizers and pesticides [14]. The N fertilizers produced through the Haber–Bosch process were crucial for food production and played a pivotal role. While the Green Revolution played an essential role in alleviating food shortages and enhancing crop production, it also established a farming model that became heavily reliant on external inputs and increasingly disconnected from natural ecological processes [15].

One of the biggest concerns stemming from this shift is the gradual decline in modern crop plants’ ability to naturally form symbiotic relationships with nitrogen-fixing microorganisms, mainly rhizobia, as reported for the legume crops soybean, pea, and alfalfa [16]. This phenomenon can also be exemplified by the development of modern wheat cultivars during the Green Revolution; although being highly productive under intensive agrochemical inputs, breeding selection inadvertently diminished the plants’ capacity to recruit and maintain complex bacterial communities in the rhizosphere, including those that indirectly contribute to N fixation [17,18]. Landraces, ancestral, and heirloom varieties often maintained these beneficial partnerships with soil bacteria and fungi, which supplied nitrogen and other compounds in exchange for carbon compounds produced during photosynthesis [19]. However, the consistent availability of mineral N decreased selective pressure for these interactions, leading to the decline and, in many cases, the loss of symbiotic capacity in modern crop varieties. Recent studies have shown that intensively fertilized agricultural systems tend to exhibit reduced diversity and functionality in the soil microbiota [20,21,22,23], thereby compromising essential ecosystem services, including nutrient cycling, water retention, and pathogen suppression.

**Figure 2:**
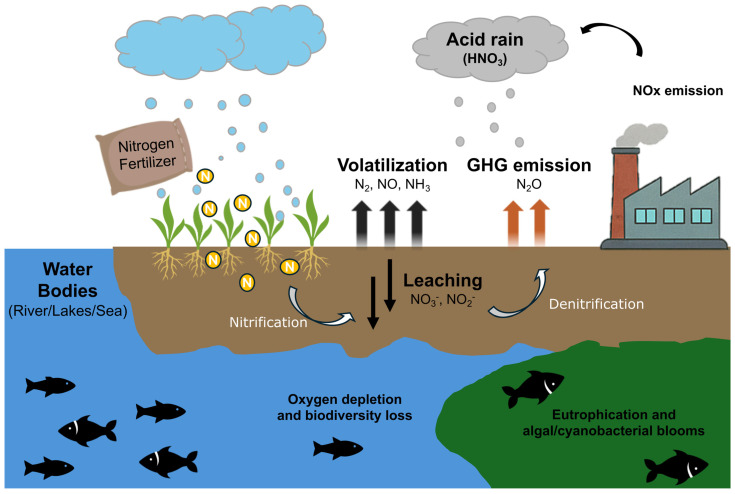
Environmental impacts of nitrogen fertilizer use and the nitrogen cycle. The figure illustrates the ecological consequences of nitrogen (N) fertilizer application. Excess N in agricultural systems leads to the volatilization of nitrogen gases (N_2_, NO, NH_3_), contributing to greenhouse gas (GHG) emissions (e.g., N_2_O) and acid rain formation (HNO_3_) through the emissions of NO, N_2_O, and NO_2_ (NOx). Nitrogen leaching in the form of nitrate (NO_3_^−^) and nitrite (NO_2_^−^) contaminates water bodies, causing eutrophication and algal blooms. This results in oxygen depletion and biodiversity loss in aquatic ecosystems. The figure highlights the need for improved NUE to mitigate these environmental impacts.

Excessive application of N fertilizers also poses significant environmental risks beyond the biological impacts (Figure 2). Elevated soluble nitrate levels contaminate water bodies, directly compromising water quality. The over-enrichment of aquatic ecosystems, commonly referred to as eutrophication, results in algal blooms and the formation of hypoxic “dead zones” in lakes, rivers, and coastal waters. The release of N as volatile gases, particularly nitrous oxide (N_2_O), a potent greenhouse gas, highly contributes to climate change and global warming potential [24]. Furthermore, repeated fertilizer applications result in soil acidification, which disrupts soil microbial activity and alters the availability of other essential nutrients [25]. Collectively, these impacts underscore the urgent need for more sustainable N management practices.

Considering these challenges, it is essential to reassess existing N management practices and adopt more sustainable strategies. These initiatives include improving Nitrogen Use Efficiency (NUE) of crop cultivars, and consequently, minimizing environmental losses while preserving or potentially increasing crop yield [26]. NUE is an established metric used to benchmark N management by considering the masses of N input over the resulting product (crop yield, biomass, etc.) [27]. The complexity of estimating NUE derives from the broader perspective that considers the diversity of soil input and available N forms, the plant-mediated responses to soil N status, the role of below-ground/root N pools, the synchrony between available N and plant N demand, crop genetics, and environment, among others [27]. NUE remains inherently limited in most agricultural systems because of the limited capacity of many crop cultivars to uptake and assimilate nitrogen efficiently, the high susceptibility of N fertilizers to losses through leaching, volatilization, and denitrification, and soil heterogeneity that constrain nutrient availability [28,29].

One promising approach involves the use of microorganisms, particularly those capable of biological nitrogen fixation (BNF), which offers an alternative complement to conventional fertilizers. These microorganisms not only supply N but also may produce phytohormones, improve stress tolerance, and support overall soil health, while assisting crop plants to enhance N uptake and assimilation capacities [29,30,31]. Approaches based on the *de novo* domestication and the use of wild relatives as genetic resources have been adopted as strategies to recover lost traits and strengthen NUE in modern crops [32,33]. Finally, innovative N delivery systems such as nano-formulated biofertilizers and controlled-release fertilizers complement this integrative approach, offering a more efficient and environmentally beneficial nutrient management solution [34].

The historical trajectory of N use in agriculture reveals a paradox: while technological advances have driven unprecedented yield increases, they have also introduced significant environmental and social trade-offs. Excess N application, inefficient N uptake by modern cultivars, and poor agronomic management practices have compromised the efficient use of N fertilization [35,36]. The challenge now is to reconcile yield with environmental responsibility by enhancing NUE through innovative and integrated approaches. This review focuses on three critical areas for improving NUE: (i) harnessing Biological Nitrogen Fixation to reduce reliance on synthetic fertilizer; (ii) understanding the molecular pathways regulating NUE in plants to optimize N acquisition and utilization; (iii) exploring novel strategies such as *de novo* domestication and nutrient release technology in fertilizers. Addressing these aspects is not just a technical challenge; it is an ethical responsibility to ensure sustainable agriculture systems and approaches for future generations.

## 2. Biological Nitrogen Fixation (BNF) for NUE

Since the provision of N for crops has been a significant challenge to agricultural development, various alternatives have been implemented as part of agricultural modernization efforts. After utilizing limited resources like guano and natural sodium nitrate, methods for supplying N to the soil through manure and cover crops were demonstrated to be more sustainable [37]. In rotational farming systems, the prior presence of a N-fixing crop affects the availability of N for subsequent crops through its natural contribution to the soil, either in assimilable forms or in organic matter. It is estimated that soybean cultivation fixes approximately 16 million tons of N year^−1^, which accounts for approximately 77% of the N fixed by cultivated legumes [38]. Furthermore, a portion of this nitrogen remains in the field following the harvest. In the case of cover legumes, such as clover, crotalaria, cowpea, and mucuna, which are incorporated into the soil, the amount of N fixed may even exceed 100 kg/ha [39,40]. Nonetheless, the contribution of BNF is not uniform and it can vary substantially with physico-chemical soil characteristics, management practices, and environmental conditions (e.g., temperature and precipitation), factors that must be considered when interpreting the contributions of this nitrogen incorporated in the soil [41,42,43,44]. The mineralization of N becomes a method for achieving the nutritional requirements of the subsequent crop, thereby reducing the need for N fertilization and, consequently, enhancing the overall efficiency of the system’s nutrient input. However, this method requires the allocation of land for cultivating N-fixing cover crops, rather than for crops designated for food.

In the context of sustainable intensification of agriculture, the utilization of microorganisms through BNF constitutes one of the most ecologically and agronomically efficient strategy for supplying N to the soil, as it does not require the use of fossil fuels. The rhizosphere, a dynamic zone surrounding plant roots, hosts a diverse community of microorganisms [45]. This root microbiome comprises bacteria, fungi, archaea, and protists interacting with plant roots through biochemical and molecular signaling pathways. These interactions modulate nutrient availability and promote plant health through N fixation, phosphorus solubilization, phytohormone production, and the suppression of pathogens in the rhizosphere [46,47].

Symbiotic nitrogen fixation (SNF) is a process that contributes to N input in natural systems through the symbiotic association between legumes and diazotrophic bacteria, primarily from the genus *Rhizobium* and related genera, including *Bradyrhizobium*, *Sinorhizobium*, and *Mesorhizobium* [48]. Microbial N fixation is responsible for approximately 122 million tons of N annually, with nearly half of the N present in agricultural soils resulting from the activity of nitrogen-fixing bacteria [49,50]. Several crops of agricultural importance directly and indirectly benefit from SNF and free-living nitrogen-fixing microorganisms, including alfalfa, beans, maize, rice, peanuts, soybeans, sugarcane, and wheat, leading to significant improvements in N availability and plant growth [51,52,53,54,55].

### 2.1. Plant-Microbe Association for N Nutrition

The symbiosis between rhizobia and legumes offers an economically feasible and environmentally friendly approach to reducing fertilizer inputs [51]. Particular species of leguminous plants form highly specialized and effective symbiotic relationships with N-fixing bacteria through nodules in their roots, where SNF occurs [48]. In these nodules, bacteria are responsible for converting N_2_ into ammonia by the nitrogenase enzyme complex, with the energetic demands supported by the host plant, which provides carbohydrates derived from photosynthesis [56]. However, relatively few plant species engage in SNF because the process is energetically costly, requiring the equivalent of 16 ATP per N_2_ reduced to ammonium, which is 36% more than the energy needed to convert nitrate to ammonium [57,58]. Consequently, nitrogen-fixing plants often downregulate SNF when soil ammonium or nitrate is readily available [56,57,59]. The crop most extensively studied for the effects of this symbiosis is soybean, where it is estimated that SNF can supply between 50% and 90% of the total N demand, depending on soil conditions, cultivars, and inoculant efficiency, saving farmers the equivalent of 60 to 200 kg of N/ha in fertilizer [38]. The inoculation of selected rhizobia strains gained widespread acceptance as an economical agronomic practice, as this approach enhances nodulation efficiency, thereby improving grain yield, particularly in N-deficient soils [60]. This practice enables emerging countries to reduce their dependence on imported N fertilizers while promoting the sustainability of large-scale grain production.

Numerous free-living N-fixing bacteria contribute to plant N nutrition [61,62]. Plant growth-promoting rhizobacteria (PGPRs) found in the rhizosphere, including *Azotobacter* and *Azospirillum*, both diazotrophic bacteria, enhance plant growth through N fixation [53,63,64]. Additionally, *Pseudomonas* and *Bacillus* species contribute to N uptake, particularly in the production of cereal and vegetable crops, leading to increased NUE [65,66,67]. Co-inoculation strategies (e.g., rhizobia + PGPR) enhance both N fixation and assimilation in crops. While rhizobia fix N, PGPRs synthesize molecules that contribute to plant growth, such as siderophores, which improve iron bioavailability, and phytohormones (e.g., auxins and gibberellins) [50,66,67]. The utilization of these beneficial microorganisms can replace between 20% and 50% of the N required for crops such as maize, rice, and sugarcane [49].

Diazotrophic cyanobacteria, such as *Anabaena*, *Calothrix*, *Nostoc*, and *Scytonema*, contribute to natural fertilization in agricultural soils by fixing N and releasing compounds that enhance soil fertility and plant growth (e.g., exopolysaccharides, vitamins, and phytohormones) [55,68]. Cyanobacterial biofertilizers are utilized in sustainable agriculture, particularly for non-leguminous crops, as they serve as a renewable source of N while promoting soil health and crop productivity [69]. The symbiosis between the cyanobacterium *Anabaena azollae* and *Azolla* sp. can fix up to 300 kg N ha^−1^ per year and has been a well-established method of fertilization as manure or a dual crop in rice paddies [70]. Additionally, the inoculation of N-fixing cyanobacteria (e.g., *Anabaena azotica* and *Tolypothrix tenuis*) can promote rice growth under saline soil conditions, decreasing oxidative stress markers, and improving amino acid content, which could be a strategy for higher-quality rice production [71].

Arbuscular mycorrhizal fungi (AMF) can establish symbiotic associations with plant roots, thereby improving the uptake of nitrate and ammonium [72,73]. AMF extend their hyphal networks into the soil, increasing the root surface area and facilitating the colonization of legume roots by symbiotic N-fixing bacteria [74]. AMF may also solubilize phosphorus, improving plant nutrition and tolerance to abiotic stresses such as drought and salinity [75]. The mutualistic relationship between AMF and host plants is mediated by complex signaling pathways, including the phytohormone strigolactone, which regulates fungal colonization and nutrient exchange [76,77]. This signal pathway is particularly important, as strigolactone is a crucial signal during stress conditions that influence rice’s responses to N deficiency and distribution to the shoots [78].

### 2.2. Factors Affecting Biological N Fixation

A complex balance of environmental and biological factors governs the regulation of SNF. The most limiting factor to SNF is oxygen availability, as nitrogenase is highly sensitive to oxygen, requiring the presence of leghemoglobin, an oxygen-scavenging molecule, to maintain a microaerophilic environment in the nodules [79,80]. A network of proteins encoded by the operons *nif* (nitrogen fixation) and *nod* (nodulation) regulates key processes such as nodulation signaling, infection thread formation, and nitrogenase activity in symbiotic bacteria [81,82,83]. These mechanisms are controlled by plant-derived flavonoids and bacterial Nod factors, ensuring specificity and efficiency in nodule formation [81,84]. SNF is highly sensitive to abiotic factors, such as soil pH, temperature, and the availability of essential micronutrients such as molybdenum (Mo) and iron (Fe), which are cofactors of the nitrogenase during the conversion of atmospheric N into ammonia [85,86,87]. Soil ammonium levels also modulate N fixation, as high concentrations of bioavailable reduced N sources can repress the expression of nitrogenase-related genes [88]. These conditions can significantly limit N fixation efficiency, affecting plant growth and yield.

The link between synthetic nitrogen fertilizer (SF) and soil health has attracted attention in recent years, as the excessive use of SF might disrupt soil microbial communities, reduce diversity, and alter natural nutrient cycles, thereby affecting soil fertility and ecosystem balance [89,90]. Different amounts of nitrogen application affect soil microbial diversity and reduce enzyme activity; therefore, the appropriate use can enhance N mineralization and nitrification, while affecting the long-term retention of nitrogen species in soils [88,91,92]. Interaction with other soil microorganisms, including mycorrhizal fungi and plant growth-promoting rhizobacteria, further impacts nodulation and N fixation efficiency [93]. Studies have shown that the field application of nanofertilizers and exogenous humic substances—modern artificial fertilizers—has a profound beneficial impact on the composition and activity of soil microorganisms responsible for N cycling in soils [94,95]. In the N cycle, microbial processes, such as nitrification and denitrification, affect N losses through leaching and gas emission (Figure 2) [96]. Nitrification is an aerobic process driven by ammonia-oxidizing bacteria (AOB) (e.g., *Nitrosomonas*, *Nitrosospira*, *Nitrosococcus*) and ammonia-oxidizing archaea (AOA) (e.g., *Nitrospira*, *Nitrososphaera*, *Nitrosopumilus*), which converts ammonium into nitrate [97,98]. In contrast, denitrification is a facultative anaerobic process, carried out by bacteria from the orders Bacillota, Actinomycetota, and Bacteroidota to reduce nitrate and nitrite to N_2_ [98,99]; nevertheless, it is also reported in aerobic bacteria such as *Pseudomonas* [100]. Soils rich in organic matter affect nitrifying and denitrifying microbial processes, which can result in significant greenhouse gas emissions, such as N_2_O [101].

Balancing nitrifying and denitrifying microbial communities through soil management practices, such as crop rotation, supplying organic matter, and reduced tillage, optimizes N availability while minimizing losses [45]. Reports indicate that AOA are more prevalent in acidic and oligotrophic soils, whereas AOB predominate in intensively managed agricultural soils with higher N inputs [97,102]. Likewise, denitrification contributes to N losses and greenhouse gas emissions, mainly in wetlands and compacted soils [97,103]. Thus, the function of soil microbial communities in N cycling further supports the use of the ecological services of microorganisms to assess beneficial soil functions (nitrogen retention and greenhouse gas mitigation) at the same time, which makes it possible to understand the N_2_O emission dynamics from different landscapes, providing feedback on land use and management [103].

### 2.3. How Can Biotechnological Approaches Improve N Fixation in the Future?

The recent discovery of the nitroplast, a N-fixing organelle in the marine alga *Braarudosphaera bigelowii*, has redefined our understanding of eukaryotic N metabolism [104]. Nitroplast derives from a cyanobacterial endosymbiont that evolved into a functional cellular compartment capable of fixing atmospheric N (N_2_). This breakthrough raises the possibility of engineering analogous organelles in crops, enabling autonomous N fixation in non-leguminous plants [105,106,107]. Prior to that, research efforts were primarily focused on engineering mitochondria for N fixation by introducing the *nif* gene cluster [108,109]. This approach was stimulated by characteristics considered beneficial for supporting nitrogenase function, like high oxygen consumption and their bacterial-type iron–sulfur cluster biosynthetic machinery in the mitochondria. Since nitrogenase is essential for N fixation, the remaining challenges to implementing this knowledge include precise gene regulation, efficient cofactor biosynthesis (e.g., FeMo-cofactor), and adequate energy supply to maintain nitrogenase activity in the host [109]. The use of nitroplast to improve NUE also presents challenges. The relatively small genome (~1.4 Mb) and the absence of several essential metabolic genes, including those encoding photosystem II, RUBISCO, and enzymes of the tricarboxylic acid cycle poses difficulties in establishing the metabolite exchange and energy flow between the nitroplast and the host plant [110]. Furthermore, the absence of these pathways remains a barrier to ensuring stable integration and successful transmission across successive plant cell generations [105,107].

Biotechnology can optimize NUE in plants, as genetic engineering has facilitated the modification of both plants and nitrogen-fixing bacteria to enhance N fixation efficiency and extend its application to non-leguminous crops. Strategies such as introducing the *nif* gene into non-leguminous plants or genetically enhancing rhizobia strains have shown potential in improving N nutrition, and reducing dependence on synthetic fertilizers [19,111]. Advances in omics tools (e.g., metagenomics and metabolomics) enabled the identification of microbial metabolic pathways that influence diverse biogeochemical processes, uncovering novel interactions between microorganisms and host plants [112]. These omics tools help to assess plants with higher nodulation capacity and to identify well-adapted rhizobia strains for various environmental conditions.

Further, genome editing methods can optimize plant-microbe interactions and symbiotic efficiency. For example, CRISPR-based approaches have been utilized to enhance flavonone biosynthesis in rice, which stimulates biofilm formation and N fixation by soil diazotrophic bacteria, resulting in increased ammonium availability and improved grain yield under conditions of limited N and aerobic stress [113]. Researchers have genetically engineered *Azorhizobium caulinodans*, *Klebsiella variicola*, *Kosakonia sacchari*, *Rhizobium* sp., and *Pseudomonas protegens* to express nitrogenase, even in the presence of synthetic fertilizers, thereby overcoming the natural regulatory mechanisms that suppress N fixation under high ammonium conditions in cereal crops, and the development of a conditional symbiosis in which the bacteria fix nitrogen only when in contact with the desired host plant [114,115,116,117]. These approaches demonstrate the potential of genome engineering for sustainable alternatives for N management in agriculture.

## 3. Molecular Pathways Regulating NUE in Plants

### 3.1. Gene Characterization

Improving NUE in plants is essential for sustainable agriculture and reducing the environmental impact of synthetic nitrogen inputs. Nitrogen availability is a key factor for plant growth, mainly through nitrate (NO_3_^−^) and ammonium (NH_4_^+^) [118]. However, these inorganic forms tend to be short-lived in soil due to leaching, microbial immobilization, and volatilization. To increase NUE, it is important to understand the molecular systems involved in N uptake, assimilation, and remobilization, including transporters, enzymes, transcriptional regulators, and signaling pathways that coordinate these processes under varying N conditions [119,120]. This section will explore first the characterization of key plant genes and transporters involved in N uptake and assimilation, followed by their functional role in improving NUE.

Plants adapt to varying N availability through dual-affinity uptake systems, enabling efficient uptake across a wide range of external N concentrations. Under these conditions, plants primarily rely on two transporter systems: the High-Affinity Transporter System (HATS), which functions under low N concentrations (<1 mM), and the Low-Affinity Transporter System (LATS), which operates at higher N concentrations (>1 mM) [118,121]. These uptake systems are primarily mediated by conserved transporter gene families: the Nitrate Transporter1/Peptide Transporter (NRT1/PTR Family or NPF), the NITRATE TRANSPORTER (NRT2), and the Ammonium Transporter (AMT) from the AMT/Mep/Rh superfamily [122,123].

The NPF family is a large transporter gene family in plants, characterized by its multifunctional transport capabilities [124]. Most of the NRT1 (NPF) family members are Low-Affinity Transporters (LATS). A notable exception is the *Arabidopsis thaliana* AtNPF6.3 (formerly AtNRT1.1), which exhibits a dual-affinity for NO_3_^−^ transport. This mechanism is regulated by phosphorylation of the Threonine-101 residue by a calcium-dependent protein kinase CIPK23, allowing the transporter to switch between low and high-affinity states [125,126,127]. Additionally, conserved residues such as Histidine-356 are critical for proton-coupled NO_3_^−^ transport, and mutations at this site disrupt transport activity [128]. Interestingly, AtNPF6.3 also affects auxin transport independently of NO_3_^−^ uptake, highlighting its multifunctionality [129]. The NRT2 family members are mostly High-Affinity Transporters (HATS) that ensure efficient uptake when external nitrate concentrations are minimal. NRT2 transporters require the cooperation of the accessory protein NRT3 (also known as NAR2) to form a stable and active transport complex for high-affinity NO_3_^−^ acquisition [130,131,132]. Nitrate transporters are predominantly expressed in roots, where they mediate NO_3_^−^ uptake from soil, while some other members are preferentially expressed in shoots, phloem, or xylem, where they contribute to the translocation and remobilization of NO_3_^−^ or other molecules such as peptides, hormones (e.g., auxin and abscisic acid), glucosinolates, and carboxylates [133,134,135,136,137,138,139]. Structurally, NRTs share a conserved 12 transmembrane-helix domain featuring a central substrate-binding cavity predominantly lined with hydrophobic residues [140,141,142].

The ammonium transporter superfamily AMT/Mep/Rh plays a central role in NH_4_^+^ transport, primarily employing NH_3_/H^+^ symport or an electrogenic uniport mechanism [143]. AMT genes are predominantly expressed in root epidermal and cortical cells, where they mediate NH_4_^+^ uptake [144,145]. Some AMT isoforms are also expressed in vascular tissue, including the xylem and phloem, where they facilitate NH_4_^+^ translocation to shoots [146,147]. AMT proteins feature a conserved 11-α-helix transmembrane domain, with a Phenylalanine gate [F(Y)XXW motif] and Aspartate-60 residue governing substrate transport [148,149]. To prevent toxic NH_4_^+^ accumulation, AMT activity is tightly regulated through post-translational modification, such as phosphorylation and ubiquitination, which inhibit or trigger protein degradation under high NH_4_^+^ conditions [150]. Beyond NH_4_^+^, AMTs transport urea/ammonia and respond to carbon status.

After uptake, inorganic N undergoes assimilation into amino acids, a process tightly regulated by enzymes and signaling pathways. Nitrate reductase (NR/NIA) initiates the process by reducing NO_3_^−^ to NO_2_^−^ using NAD(P)H and a molybdenum cofactor. Nitrite reductase (NIR) then reduces NO_2_^−^ to NH_4_^+^ in plastids [151]. This step is regulated by light and carbon availability [152]. The NH_4_^+^ then is fed into the GS/GOGAT cycle, where glutamine synthetase (GS) combines NH_4_^+^ with glutamate to form glutamine, and glutamate synthase (GOGAT) regenerates glutamate using α-ketoglutarate [126].

This cycle operates across compartments; the cytosolic GS1 mobilizes N for remobilization and translocation to the shoot [153], while the plastid-located GS2 plays a crucial role in primary NH_4_^+^ assimilation. Cytosolic GS1 exhibits up to 9.5-fold induction under NH_4_^+^ dominance compared to plastidic GS2, emphasizing isoform-specific functions [154]. These enzymatic processes are critical for integrating N into the plant’s metabolic framework, ensuring efficient utilization and minimizing waste.

To learn more about these pathways during N assimilation and remobilization, please refer to other reviews [155,156].

### 3.2. Functional Roles in Improving NUE

Progress in understanding gene function and molecular interactions has enabled targeted genetic and biotechnological interventions to optimize N acquisition and metabolism, thereby improving NUE. Despite important progress, several limitations continue to challenge the translation of these findings into NUE improvement. This section explores some key findings and their implications for enhancing NUE in plants.

In rice (*Oryza sativa*), several transporters have been identified that significantly impact NUE and grain yield. For example, OsNLP3.1, a low-affinity transporter, regulates NO_3_^−^ allocation to shoots, reducing N loss, and a point mutation (S82F) increases NUE, biomass, and grain yield under low-N conditions [157]. Similarly, OsPTR9 (OsNPF8.9a), which mediates NO_3_^−^ and dipeptide transport, increases NO_3_^−^ translocation to shoots, and its overexpression boosts grain yield and NUE [158]. OsNPF7.3, localized at the vacuolar membrane and induced by organic N, contributes to N allocation during grain filling and increases yield under N limitation [159]. Furthermore, *OsPTR8.9a*, a low-affinity transporter, when co-overexpressed with *OsNR2*, significantly increased N uptake, resulting in higher grain yield [160].

Substantial contributions by the activity of specific transporters to NUE have been demonstrated in other species. In grapevine (*Vitis vinifera*), the high-affinity transporter VvNPF6.5, localized in the plasma membrane, increased N content and NUE under N-deficient conditions when ectopically expressed in *A. thaliana* [161]. Similarly, in eucalyptus (*Eucalyptus grandis*), *EgNPF4.1* and *EgNPF7.3* exhibit root-specific induction under N limitation, suggesting their roles in vascular loading for N allocation [162]. These findings highlight the conserved yet species-specific roles of transporters in optimizing N uptake and loading.

NRT2 transporters play a crucial role in enhancing NUE under low-N conditions. In *A. thaliana*, AtNRT2.1 and AtNRT2.2 increased NO_3_^−^ uptake efficiency at low N levels, with NRT2.1 regulating root system architecture to boost N foraging [163,164]. AtNRT2.4 is functionally active in the epidermis under severe N starvation, maintaining N supply for growth and metabolism [165]. In cereal, orthologous transporters like OsNRT2.3b in rice and ZmNRT2.1 and ZmNRT2.2 in maize increase NO_3_^−^ absorption and internal N remobilization, with OsNRT2.3b supporting phloem loading and long-distance transport [166,167]. Overexpression of *OsNRT2.3b* significantly increases grain yield and NUE in rice [167]. Similarly, *TaNRT2.1* and *TaNRT2.2* in wheat contribute to NO_3_^−^ acquisition under low N field conditions, correlating with agronomic NUE [133]. In *Brassica napus*, low N stress induces root system modification and increased plasma membrane H^+^-ATPase activity, improving NO_3_^−^ uptake [168]. Additionally, in *B. napus*, AUX-1-mediated root morphology adjustments and differential regulation of NRTs, such as upregulation of *BnNRT1.5* and downregulation of *BnNRT1.8*, led to an increase in N allocation to aerial tissues [168].

Research on AMTs has revealed their critical role in enhancing NUE. In rice, overexpression of *OsAMT1;2* increased NH_4_^+^ uptake under low-N conditions, leading to improved N recovery and increased grain yield [169]. Similarly, *BrAMT1;5* from Chinese cabbage (*B. rapa*), when overexpressed in *A. thaliana*, boosted NH_4_^+^ influx, improved biomass, and root elongation under low-N conditions [170].

The synergistic effects of co-overexpressing multiple genes involved in N metabolism have also been demonstrated. In rice, lines co-overexpressing *OsAMT1;2*, *OsGS1;2*, and *OsAS1* (asparagine synthetase) increased NUE and improved grain yield compared to single-gene transgenic [160]. In maize, *ZmAMT1;3* and the *ZmNRT2;5* coordinate N uptake, improving NUE under N limitation and drought stress [171]. Similarly, in sorghum (*Sorghum bicolor*), *SbAMT3;1* enhances root NH_4_^+^ uptake under drought conditions, increasing N uptake [172]. In sugarcane (*Saccharum* spp.), *ScAMT3;3*, a member of the AMT2 subfamily, has been characterized as a low-affinity transporter. Functional studies in yeast and *A. thaliana* confirmed its role in NH_4_^+^ transport and its presumed involvement in NH_4_^+^ remobilization via vascular loading, which might contribute to improved NUE and yield [147].

Synergistic interactions between transporters further underscore their importance in improving NUE. For example, co-overexpression of *OsNPF8.9* and *OsAMT1;2* in rice has been shown to enhance root architecture and transport kinetics, resulting in an increase in yield under low N conditions, through an improved root system [160]. Additionally, increased transcript abundance of Nitrate Reductase (NR) in rice correlates with an improvement in NUE under low-N conditions [173]. However, constitutive overexpression of transporters such as *OsAMT1;2* can lead to N toxicity, demanding a refined expression strategy, such as the use of tissue-specific promoters or co-expression with *OsGOGAT1* to balance N uptake and assimilation [169].

Species-specific adaptations and environmental factors further complicate efforts to optimize NUE. For instance, in *Betula platyphylla* (white birch), seedlings from different geographical provenances exhibit distinct N uptake kinetics and preferences, reflecting their adaptation to local N regimes [174]. Similarly, in sugarcane (*Saccharum* spp.), genotypes with contrasting NUE display differential N form preference, where inefficient genotypes exhibit lower NH_4_^+^ uptake capacity compared to efficient ones, suggesting a strong link between N form preference and overall NUE [175,176]. In *Hydrocotyle verticillate*, genotypic diversity within populations enhances NH_4_^+^ uptake rates and alters biomass allocation mediated by changes in soil nitrogen pools and potential interactions with root symbionts [177]. These findings underscore the complexity of NUE optimization, as species and genotypes adapt their N uptake strategies to specific ecological and environmental contexts.

Further illustrating these adaptations, duckweed universally favors NH_4_^+^ as its primary inorganic N source [178], while strawberries exhibit a preference for NO_3_^−^ uptake [179]. These differences underscore the need for a tailored approach to NUE improvement, based on species and environmental context. Moreover, field validation remains critical, as most data are derived from controlled environments. For example, in *Brassica napus*, field trials have shown that NUE gains diminish at N application rates exceeding 120 kg N/ha, highlighting the importance of optimizing fertilizer use in agricultural field conditions [180]. Species-specific adaptations also extend to AMT functionality. In *A. thaliana*, *AtAMT1.1* and *AtAMT1.3* are prioritized in expression, whereas in rice, *OsAMT1.1* and *OsAMT3.1* are expressed in flooded paddies, reflecting adaptations to distinct ecological niches [181,182].

Enzymatic pathways also play a crucial role in improving NUE. Overexpression of *OsGS1;2*, a key enzyme in the GS/GOGAT cycle, has been shown to elevate glutamine synthesis, thereby increasing grain protein content under reduced N fertilizer input [160]. Additionally, balancing NH_4_^+^ and NO_3_^−^ has been demonstrated to optimize N assimilation. For instance, integrated ammonium-nitrate nutrition increases soil microbial biomass N by 60% and upregulates *GS2* expression in rice roots, enhancing NUE [183].

Transcription factors act as central regulators of N signaling and metabolism. The NLP (NIN-Like Protein) family is a master regulator of the N response. In barley, *HvNLP2* knockout mutants exhibit a 40–50% decrease in the expression of nitrate uptake genes (*NRT2.1* and *NRT2.5*), and mutant plants experience severe chlorosis under low-N conditions [184]. In rice, *OsNLP4* integrates N-Fe homeostasis, increasing tiller number and yield through coordinated expression of *OsAMT1;2* and ferritin genes [185]. Similarly, *Dof1* (DNA-binding with One Finger) facilitates the provision of carbon skeletons for amino acid synthesis, with overexpression of wheat *TaDof1* increasing GS activity by 1.8-fold and grain yield by 12% under N stress [186].

Hormonal crosstalk further integrates N signaling with plant development. In rice, auxin biosynthesis inhibition by *DNR1* (*DULL NITROGEN RESPONSE1*) reduces lateral root initiation, impairing N uptake. However, cytokinin-mediated *SOD5* (*SUPPRESSOR OF DR4*) enhances auxin accumulation, rescuing NUE by 35% in *dnr1* knockout mutants [187]. Additionally, remobilization processes are critical for efficient N use. For example, *OsATG8b/c* activates autophagy under N starvation, remobilizing 40% more N from senescing leaves to grains [188]. Similarly, phloem-specific *OsNRT1.7* facilitates remobilization during senescence, improving N recovery by 18–25% in rice [189,190].

Despite significant progress, challenges remain, particularly in understanding the interaction of transcription factors, hormonal pathways, and species-specific differences in key transporter gene families such as NRT (NPF and NRT2), and AMT [191]. Comparative analyses reveal that while NRT (NPF and NRT2) gene counts are generally comparable between monocots and dicots, dicots show a higher number of AMT genes than monocots (Table 1). These patterns are influenced by factors such as polyploidy, genome size, and the quality of genome annotation, complicating broad cross-species applications and generalizations. This underscores the importance of adopting lineage-specific approaches. Furthermore, genetic diversity and environmental variability continue to hinder the practical application of molecular insights into sustainable agricultural solutions [192]. Future research should focus on unraveling the genetic and environmental interactions unique to each plant lineage, enabling the development of targeted strategies by genetic manipulation to enhance NUE.

## 4. Novel Approaches for Improving NUE

### 4.1. De Novo Domestication

*De novo* domestication represents a modern molecular approach that rapidly converts wild or semi-domesticated plants into domesticated forms by editing specific genes or alleles. This approach aims to retain valuable alleles often lost during crop domestication and/or breeding, responsible for traits associated with, for instance, high NUE and stress resilience, but targets key domestication syndrome traits, such as plant architecture, fruit and seed size, seed shattering, and dormancy, which are essential for agricultural viability [223,224]. By addressing these traits, *de novo* domestication ensures that wild genotypes are transformed into semi-agronomically suitable crops while maintaining their inherent advantages. In contrast to traditional domestication that spans centuries or millennia, *de novo* domestication instead leverages comprehensive genomic information and tools, like CRISPR/Cas9 (Clustered Regularly Interspaced Short Palindromic Repeats—CRISPR-Associated Protein 9), to achieve targeted improvements within years [225]. The premise is to bypass the genetic bottlenecks and drift associated with traditional domestication/breeding, which often leads to the loss of valuable alleles for resilience and nutritional value [226,227].

The process is initiated by identifying promising accessions from wild relatives or novel species that exhibit robust N uptake, assimilation, and remobilization abilities under diverse stress conditions [226]. Subsequent genetic improvement employs integrated approaches: conventional breeding can introduce NUE-related genes; genetic modification can insert regulatory elements, such as transcription factors, and gene editing (e.g., CRISPR-Cas9) can precisely edit regulatory regions or genes to enhance NUE without introducing undesirable traits by linkage drag [228,229,230]. This multifaceted approach offers significant potential for improving NUE across diverse plant systems.

*De novo* domestication has been demonstrated to improve NUE in various crops. In foxtail millet (*Setaria* spp.), comparative analysis between wild and domesticated genotypes under variable N regimes revealed genetic variations in NUE. These findings, based on field-based physiological and phenotypic evaluation, underscore the impact of domestication on root architecture and response to nitrogen. This domestication-driven shift offers valuable insights for developing targeted breeding strategies [227]. Similarly, perennial plants adapted to nutrient-poor soils minimize N losses through efficient nutrient retention and recycling while serving as models for identifying key genes linked to NUE [223]. Insights from cotton domestication suggest that optimizing photosynthetic NUE (PNUE) through improved N allocation to photosynthetic machinery provides a transferable principle for other crops [224]. Enhancing below-ground traits, such as root architecture, represents a promising pathway to increase N uptake efficiency and overall NUE [231]. In *Silphium integrifolium*, comparing wild and improved accessions reported two to three-fold increases in biomass and N acquisition, with detailed quantification of N allocation, and internal cycling [232]. Similarly, *Setaria viridis*, a wild C4 grass and a model species for Panicoideae domestication, has emerged as a promising platform for NUE improvements. High-quality genomic resources and the identification of agronomically relevant loci, such as the *SvLes1* gene controlling seed shattering, have enabled the recreation of domesticated alleles through CRISPR-based editing [233]. In cotton, multiplex editing of domestication syndrome genes has enhanced fruit size and yield under N-limited conditions without introducing undesirable traits through linkage drag [234]. Advances in tomato genomics and gene editing have revealed the central role of gene interactions in quantitative trait variation. By manipulating regulatory genes such as *Self-Pruning* (*SP*), *Singles Flower Truss* (*SFT*), *Clavata3* (*CLV3*), and *Wuschel* (*WUS*), researchers have successfully modulated plant architecture, productivity, and flowering time, demonstrating the potential of epistasis-driven approaches to accelerate domestication and improve NUE [235].

The integration of advanced genomic genetic tools, such as CRISPR-based editing and prime editing, has further expanded the scope of *de novo* domestication. For instance, a novel pipeline for domesticating abiotic stress-tolerant wild plants emphasizes the recovery of lost tolerance traits through genetic editing. This approach, combined with reference genome, FIGS (Focused Identification of Germplasm Strategy), and speed breeding, offers a promising solution for developing resilient crops [236]. Additionally, introgression of genomic regions from *Solanum pimpinellifolium* into cultivated tomato has conferred greater resilience to N deficiency, with specific alleles linked to genes such as *Glutamine Synthetase 1* (*GS1*), *Sucrose Phosphate Phosphatase* (*SPP*), and *Invertase* (*INV*) maintaining productivity and biomass. These findings highlight the potential of wild alleles in developing sustainable cultivars [237].

Advancements in omics technologies have revolutionized the identification and manipulation of NUE-related traits. Next-generation sequencing has enabled high-resolution mapping of quantitative trait loci (QTLs) and candidate genes associated with NUE. The QTL-seq approach has been successfully applied to crops like rice, soybean, and tomato to identify loci governing traits such as seed dormancy and yield components [238,239]. RNA sequencing (RNA-seq) has facilitated the identification of gene expression patterns under stress conditions, while proteomics and metabolomics have provided insight into biochemical pathways regulating NUE. For instance, metabolite profiling in teosinte has uncovered genes linked to grain yield and morphology in maize, offering avenues for metabolic engineering [240]. CRISPR/Cas9 genome editing has further accelerated the domestication of wild species. In tomato, editing genes such as *SP* and *CLV3* has improved fruit morphology and lycopene content within a single generation [33]. Similarly, in wild rice *Oryza alta*, multiplex editing of genes like *OaSD1* (*Semi-Dwarf 1*) and *OaAn-1* (*Awn Length Regulator 1*) has enhanced yield, stress tolerance, and tolerance to nutrient deficiency [241]. These breakthroughs highlight the transformative potential of omics-guided gene editing for sustainable crop improvement.

Balancing these multiple, often conflicting traits, remains a central hurdle in *de novo* domestication efforts [228]. NUE is a polygenic trait influenced by genotype-environment interactions, making it difficult to optimize without trade-offs. Enhancing N partitioning between roots and shoots may improve NUE but could compromise yield or disease resistance. Balancing these conflicting traits requires meticulous management and a deep mechanistic understanding of NUE pathways [242,243]. Despite the complexity of NUE improvement, the integration of multiple approaches, including conventional breeding, genetic modification, and gene editing, needs to be combined to achieve sustainable improvements [119]. *De novo* domestication offers a short-term strategy for sustainable agriculture by integrating advanced genetic tools with omics technologies to develop new genotypes that require less N fertilizer, thereby reducing environmental impacts and improving system sustainability [244,245]. When combined with near-term solutions, such as precision nutrient management, *de novo* domestication accelerates the building of a resilient agricultural system that can meet global food demands while preserving ecological integrity [246,247,248].

*De novo* domestication represents a paradigm shift in crop breeding, enabling the rapid transformation of wild species into agriculturally viable crops with enhanced NUE. By leveraging genomic resources, gene editing techniques, and omics-guided approaches, these strategies address the limitations of traditional breeding while unlocking the potential of wild domestication, holding the promises of revolutionizing agriculture, ensuring food security, and mitigating the environmental impacts of intensive farming practices [249,250,251,252].

### 4.2. Nutrient Release

Recent advances in modern fertilizer technologies offer complementary strategies to further optimize nutrient availability and sustainability, beyond balanced mineral and organic fertilization and other farming practices (e.g., crop rotation and irrigation) that play a key role in enhancing NUE under field conditions [253,254,255]. Traditional N fertilizers suffer from substantial losses through leaching, volatilization, and denitrification, which negatively impact crop NUE and cause environmental contamination [256]. Slow-release fertilizers (SRFs) and coated fertilizers address these challenges by synchronizing nutrient release with plant demand, minimizing losses, and enhancing uptake [257]. SRFs employ mechanisms such as diffusion, dissolution, and microbial degradation, whereas coated fertilizers utilize physical barriers, including sulfur, polymers, or bio-based materials like lignin and chitosan, to mediate the release rates [258,259]. For instance, sulfur-coated urea and polymer-coated urea reduce N losses compared to conventional urea, and they can further mitigate greenhouse gas emissions and ammonia volatilization by integrating cover crops with SRFs [260,261]. However, environmental factors such as temperature and moisture, along with high SRF production costs, limit their scalability [257].

The controlled release of nutrients constitutes a significant advantage of nanofertilizers (NFs), an approach that utilizes nanoscale particles to improve crop NUE while mitigating environmental impacts [262]. NFs that use materials such as chitosan, zinc oxide, and nanocomposites can improve plant uptake due to their small size (typically <100 nm), resulting in a high surface area-to-volume ratio that increases solubility, reactivity, and interaction with plant root surfaces [263,264]. Chitosan-NPK nanoparticles have been demonstrated to improve nutrient uptake and provide a sustained supply of nutrients, thereby reducing nutrient loss and optimizing plant uptake [259,265]. ZnO nanoparticles strengthen photosynthetic activity, promote plant growth, and positively influence the microbial community structure of plants, while increasing nitrogen content [264,266,267,268]. The controlled-release properties of NFs improve plant growth, soil health, water retention, and stress resilience, while helping to reduce environmental losses through nitrogen leaching and volatilization [269,270,271]. However, the potential phytotoxicity of nanoparticles or toxicity to ecosystems, human and animal health, demands further research to ensure long-term safety [272].

Innovative materials, primarily those derived from biomass, reduce environmental impacts and are shaping the future of nutrient release technologies. Lignin–montmorillonite biocomposites and biochar-based SRFs, such as rice straw bio-urea, offer sustainable alternatives that enhance soil health, improve N retention, and store carbon, thereby promoting the circularization of agricultural residues [258,273]. Furthermore, urea–kaolinite–chitosan composites and nitro-humic fertilizers derived from lignite waste further enhance release profiles and agronomic efficiency, with nitro-humic acid fertilizer increasing crop NUE by 36.2% [269,273,274,275]. The development of smart fertilizers, responsive to environmental cues, and multifunctional nanofibers that combine nutrient delivery with other needs (e.g., pest and disease control) holds transformative potential in agriculture [269,276,277]. Nevertheless, comprehensive life cycle assessments are crucial for evaluating sustainability and ensuring these advancements can be scaled to meet global agricultural demands.

## 5. Conclusions

Improving NUE is not just an agricultural challenge but a global ecological priority that requires the groundbreaking integration of biotechnology, microbiology, and materials science. Key advances, including microbial consortia to reduce fertilizer dependency, CRISPR-edited genes to enhance crop performance under low-N conditions, and nano-fertilizers for synchronized nutrient release, represent strides toward sustainable agriculture. Transformative opportunities, including engineered nitroplast for autonomous N fixation in cereals and *de novo* domestication to recover lost NUE traits from wild relatives, hold immense potential to reduce reliance on synthetic fertilizer and mitigate environmental impacts. Our synthesis emphasizes that, despite these innovations, their applications remain restricted to laboratories or pilot studies owing to systemic barriers such as inadequate policy incentives for sustainable nitrogen management, insufficient funding for field validation of biotechnological advancements, and the continued undervaluation of soil microbial health within conventional agricultural systems. Moreover, while we highlight promising technologies, uncertainties remain regarding their economic feasibility, scalability, and potential unintended ecological effects. Addressing these gaps requires a coordinated and urgent effort.

To avert further ecological degradation, we propose three actions: (1) scaling up microbial and nano-based solutions to ensure their widespread adoption; (2) accelerating the implementation of biotech innovation, such as nitroplast crops; and (3) introducing regulatory mechanisms that internalize the environmental costs of N losses, such as pricing systems for N_2_O emissions or other N losses. Without a unified approach that prioritizes speed, integration, and accountability, humanity risks exceeding planetary N boundaries, jeopardizing long-term food security and ecological stability.

## Figures and Tables

**Table 1:** Abbreviated list of published Nitrate Transporter—NRT (NPFs and NRT2s) and Ammonium Transporter (AMT) family gene counts across selected monocot and dicot crop plant species. Mean values are provided for each group, with the bottom row displaying the average monocots-to-dicots ratios.

Species	Total NRT *	Total AMT	Reference
Monocots			
*Oryza sativa* (rice)	97	8	[193,194,195]
*Zea mays* (maize)	85	12	[196,197,198]
*Hordeum vulgare* (barley)	41	7	[199]
*Saccharum* spp. (sugarcane)	198	7	[147,200,201,202]
*Triticum aestivum* (wheat)	77	8	[203]
Mean	99.6	8.4	
Dicots			
*Arabidopsis thaliana* (thale cress)	60	6	[182,204]
*Glycine max* (soybean)	125	16	[205,206]
*Brassica rapa* (Chinese cabbage)	107	20	[207,208,209]
*Brassica napus* (rapeseed)	210	26	[208,209,210]
*Nicotiana tabacum* (tobacco)	148	9	[211,212,213]
*Solanum lycopersicum* (tomato)	89	5	[214,215,216]
*Populus trichocarpa* (poplar)	74	16	[123,217]
*Malus domestica* (apple)	82	15	[218,219]
*Spinacia oleracea* (spinach) **	66	-	[220]
*Solanum tuberosum* (potato) **	81	-	[221,222]
*Poncirus trifoliata* (trifoliate orange) **	62	-	[141]
Mean	100.4	14.1	
Mean Monocots:Dicots NRT/AMT Number of Genes Ratio	0.99	0.6	

* NRT represents the sum of genes from the Nitrate Transporter1/Peptide Transporter Family (NPF) and NRT2 families. ** Species with insufficient AMT data (unavailable, incomplete, or ambiguous).

## Data Availability

All the data discussed are provided in the article.
